# Surveillance of Waterborne Disease Outbreaks Associated with Drinking Water — United States, 2015–2020

**DOI:** 10.15585/mmwr.ss7301a1

**Published:** 2024-03-14

**Authors:** Jasen M. Kunz, Hannah Lawinger, Shanna Miko, Megan Gerdes, Muhammad Thuneibat, Elizabeth Hannapel, Virginia A. Roberts

**Affiliations:** ^1^Division of Foodborne, Waterborne, and Environmental Diseases, National Center for Emerging and Zoonotic Infectious Diseases, CDC; ^2^Chenega Corporation, Atlanta, Georgia; ^3^Division of Bacterial Diseases, National Center for Immunization and Respiratory Diseases, CDC

## Abstract

**Problem/Condition:**

Public health agencies in U.S. states, territories, and freely associated states investigate and voluntarily report waterborne disease outbreaks to CDC through the National Outbreak Reporting System (NORS). This report summarizes NORS drinking water outbreak epidemiologic, laboratory, and environmental data, including data for both public and private drinking water systems. The report presents outbreak-contributing factors (i.e., practices and factors that lead to outbreaks) and, for the first time, categorizes outbreaks as biofilm pathogen or enteric illness associated.

**Period Covered:**

2015–2020.

**Description of System:**

CDC launched NORS in 2009 as a web-based platform into which public health departments voluntarily enter outbreak information. Through NORS, CDC collects reports of enteric disease outbreaks caused by bacterial, viral, parasitic, chemical, toxin, and unknown agents as well as foodborne and waterborne outbreaks of nonenteric disease. Data provided by NORS users, when known, for drinking water outbreaks include 1) the number of cases, hospitalizations, and deaths; 2) the etiologic agent (confirmed or suspected); 3) the implicated type of water system (e.g., community or individual or private); 4) the setting of exposure (e.g., hospital or health care facility; hotel, motel, lodge, or inn; or private residence); and 5) relevant epidemiologic and environmental data needed to describe the outbreak and characterize contributing factors.

**Results:**

During 2015–2020, public health officials from 28 states voluntarily reported 214 outbreaks associated with drinking water and 454 contributing factor types. The reported etiologies included 187 (87%) biofilm associated, 24 (11%) enteric illness associated, two (1%) unknown, and one (<1%) chemical or toxin. A total of 172 (80%) outbreaks were linked to water from public water systems, 22 (10%) to unknown water systems, 17 (8%) to individual or private systems, and two (0.9%) to other systems; one (0.5%) system type was not reported. Drinking water-associated outbreaks resulted in at least 2,140 cases of illness, 563 hospitalizations (26% of cases), and 88 deaths (4% of cases). Individual or private water systems were implicated in 944 (43%) cases, 52 (9%) hospitalizations, and 14 (16%) deaths.

Enteric illness-associated pathogens were implicated in 1,299 (61%) of all illnesses, and 10 (2%) hospitalizations. No deaths were reported. Among these illnesses, three pathogens (norovirus, *Shigella*, and *Campylobacter)* or multiple etiologies including these pathogens resulted in 1,225 (94%) cases. The drinking water source was identified most often (n = 34; 7%) as the contributing factor in enteric disease outbreaks. When water source (e.g., groundwater) was known (n = 14), wells were identified in 13 (93%) of enteric disease outbreaks.

Most biofilm-related outbreak reports implicated *Legionella* (n = 184; 98%); two nontuberculous mycobacteria (NTM) (1%) and one *Pseudomonas* (0.5%) outbreaks comprised the remaining. *Legionella*-associated outbreaks generally increased over the study period (14 in 2015, 31 in 2016, 30 in 2017, 34 in 2018, 33 in 2019, and 18 in 2020). The *Legionella*-associated outbreaks resulted in 786 (37%) of all illnesses, 544 (97%) hospitalizations, and 86 (98%) of all deaths. *Legionella* also was the outbreak etiology in 160 (92%) public water system outbreaks. Outbreak reports cited the premise or point of use location most frequently as the contributing factor for *Legionella* and other biofilm-associated pathogen outbreaks (n = 287; 63%). *Legionella* was reported to NORS in 2015 and 2019 as the cause of three outbreaks in private residences (*2*).

**Interpretation:**

The observed range of biofilm and enteric drinking water pathogen contributing factors illustrate the complexity of drinking water-related disease prevention and the need for water source-to-tap prevention strategies. *Legionella*-associated outbreaks have increased in number over time and were the leading cause of reported drinking water outbreaks, including hospitalizations and deaths. Enteric illness outbreaks primarily linked to wells represented approximately half the cases during this reporting period. This report enhances CDC efforts to estimate the U.S. illness and health care cost impacts of waterborne disease, which revealed that biofilm-related pathogens, NTM, and *Legionella* have emerged as the predominant causes of hospitalizations and deaths from waterborne- and drinking water-associated disease.

**Public Health Action:**

Public health departments, regulators, and drinking water partners can use these findings to identify emerging waterborne disease threats, guide outbreak response and prevention programs, and support drinking water regulatory efforts.

## Introduction

Access to and provision of safe water in the United States is critical to protecting public health (*1*). Disruptions to water service caused by drinking water contamination can negatively impact public health and erode public trust in drinking water quality. Each year in the United States, waterborne pathogens cause an estimated 7.15 million illnesses, 118,000 hospitalizations, and 6,630 deaths, resulting in $3.33 billion in direct health care costs (*2*). Drinking water exposures are associated with 40% of hospitalizations and 50% of deaths and are primarily linked with biofilm pathogens such as *Legionella* and nontuberculous mycobacteria (NTM), costing the United States $1.39 billion annually (*3*). Biofilms are microbial communities that attach to moist surfaces (e.g., water pipes) and provide protection and nutrients for many different types of pathogens, including *Legionella* and NTM (*3*,*4*). Biofilm can grow when water becomes stagnant or disinfectant residuals are depleted, resulting in pathogen growth (*3*). Furthermore, biofilm pathogens are difficult to control because of their resistance to water treatment processes (e.g., disinfection) (*3*). Exposure to biofilm pathogens can occur through contact with, ingestion of, or aerosol inhalation of contaminated water from different fixtures (e.g., showerheads) and devices (e.g., humidifiers) (*3*).

Public health surveillance and other prevention programs support water treatment, regulations, and building or household water management practices in reducing waterborne diseases. Public health agencies in the United States, District of Columbia, Federated States of Micronesia, Guam, Marshall Islands, Northern Mariana Islands, Palau, Puerto Rico, and U.S. Virgin Islands investigate and can voluntarily report waterborne disease outbreaks to CDC through the National Outbreak Reporting System (NORS) (https://www.cdc.gov/nors/about.html).

This report summarizes data on drinking water-associated outbreaks reported to NORS during 2015–2020. Drinking water, also called tap or potable water, includes water collected, treated, stored, or distributed in public and individual water systems or commercially bottled and distributed for individual use. Drinking water is used for consumption and other domestic uses (e.g., drinking, bathing, showering, handwashing, food preparation, dishwashing, and maintaining oral hygiene). This report summarizes outbreak contributing factors (i.e., practices and factors that lead to outbreaks) and, for the first time, categorizes outbreaks as biofilm pathogen or enteric illness associated (*5*–*9*). Public health departments, regulators, and drinking water partners can use the findings in this report to guide outbreak response and prevention programs and drinking water regulatory efforts.

## Methods

### Data Source

CDC’s Waterborne Disease and Outbreak Surveillance System began in 1971, with reporting via paper forms through 2008. CDC launched NORS (https://www.cdc.gov/nors/about.html) in 2009 as a web-based platform for state, local, and territorial health departments to enter reports of all waterborne and foodborne disease outbreaks and all enteric disease outbreaks resulting from transmission by contact with contaminated environmental sources, infected persons or animals, or unknown modes. Most outbreaks in the United States are investigated by state, local, and territorial health departments. Outbreak information is then voluntarily reported to CDC by the public health agency that conducted the investigation. CDC might be involved in outbreak investigations that involve more than one state, are particularly large, or for which the state or local health department requests assistance.

### Waterborne Outbreak Definitions and Specifications

NORS users enter a confirmed or suspected etiology, if known, including species, serotype, or other characteristics. Etiologies for reported drinking water outbreaks in NORS can include infectious (e.g., *Campylobacter*, *Cryptosporidium*, *Giardia*, and *Legionella*) and noninfectious (e.g., copper and nonbacterial toxins) agents.

For NORS reporting, an outbreak is defined as two or more cases of similar illness associated with a common exposure. Outbreaks reported to NORS must include two or more cases linked epidemiologically by time, location of water exposure, and illness characteristics; the epidemiologic evidence must implicate water exposure as the probable source of illness for an event to be defined as a waterborne disease outbreak. Premise plumbing refers to a building’s hot- and cold-water piping system. For this analysis, the premise plumbing pathogens NTM, *Pseudomonas*, and *Legionella* were defined as biofilm-associated pathogens (*2*,*10*). In addition, consistent with the literature, *Campylobacter*, *Cryptosporidium*, *Giardia*, norovirus, and *Shigella* were defined as infectious enteric pathogens (*2*,*11*). Outbreaks of unknown etiology and noninfectious illness (e.g., chemical or toxin), were classified in their own categories.

Community and noncommunity water systems are defined by the Environmental Protection Agency as public water systems that have ≥15 service connections or serve an average of ≥25 residents for ≥60 days per year. A community water system serves year-round residents of a community, subdivision, or mobile home park. A noncommunity water system serves an institution, industry, camp, park, hotel, or business and can be nontransient or transient. Nontransient systems serve ≥25 of the same persons for ≥6 months of the year but not year-round (e.g., factories and schools), whereas transient systems provide water to places in which persons do not remain for long periods (e.g., restaurants, highway rest stations, and parks). Individual water systems are small systems not owned or operated by a water utility that have <15 connections or serve <25 persons (*12*).

NORS encourages users to indicate, when known, the water source and water source description for drinking water outbreaks. Water sources for drinking water outbreaks listed in NORS include groundwater, surface water, groundwater under the influence of surface water, other, and unknown. Water source descriptions listed in NORS include lake or reservoir, ocean, pond, river or stream, spring, well, other, and unknown.

NORS defines water treatment as the treatment usually provided before water use or water consumption, regardless of whether these treatments were operating correctly at or just before the time of the outbreak. Possible water treatment methods listed for the drinking water systems in NORS include disinfection, filtration, coagulation, flocculation, no treatment, other, and unknown.

NORS includes options to indicate where the exposure to water occurred. Settings of exposure for drinking water outbreaks listed in NORS include apartment or condominium; assisted living or rehabilitation facility, camp or cabin setting; community or municipality; hospital or health care facility; hotel, motel, lodge, or inn; long-term care facility; mobile home park; private residence; resort, restaurant or cafeteria; school, college, or university; subdivision or neighborhood; and several other types of settings.

NORS encourages users to indicate which, if any, contributing factors led to the outbreak. Users can select contributing factors from a list related to drinking water outbreaks or enter their own factors. Contributing factors for this reporting period included documented or observed (if information is gathered during document reviews, direct observations or interviews) or suspected (if factors that might have occurred but for which no documentation or observable evidence is available). Contributing factors were categorized by factor types including source (water quality was affected by a problem occurring with the source water), treatment (water quality was affected by a problem occurring with water treatment), distribution (water quality was affected by a problem within the distribution system before entry into a building or house), and premise or point of use (water quality was affected by a problem after the water meter or outside the jurisdiction of the public water utility).

### Data Analysis

CDC analyzed outbreaks reported in NORS as of October 18, 2022, (via CDC 52.12 Form) to provide information about drinking water-associated waterborne disease outbreaks in the United States in which the first illness occurred during 2015–2020. For each outbreak, NORS users provided data (when known) about: 1) number of cases, hospitalizations, and deaths; 2) etiologic agent (confirmed or suspected); 3) implicated water system and treatment method; 4) setting of exposure (e.g., hospital or health care facility; hotel, motel, lodge, or inn; and private residence); and 5) relevant epidemiologic and environmental data needed to understand the outbreak occurrences and contributing factor classification.

CDC calculated descriptive statistics on characteristics of reported drinking water outbreaks. Data cleaning, management, and analysis were conducted using SAS (version 9.4; SAS Institute) and Microsoft Excel for Microsoft 365 Microsoft Office (version 2022; Microsoft Corporation). The analysis included both confirmed and suspected etiologies. Outbreaks with multiple etiologies were classified and analyzed as one outbreak.

## Results

### All Outbreaks

**FIGURE 1:**
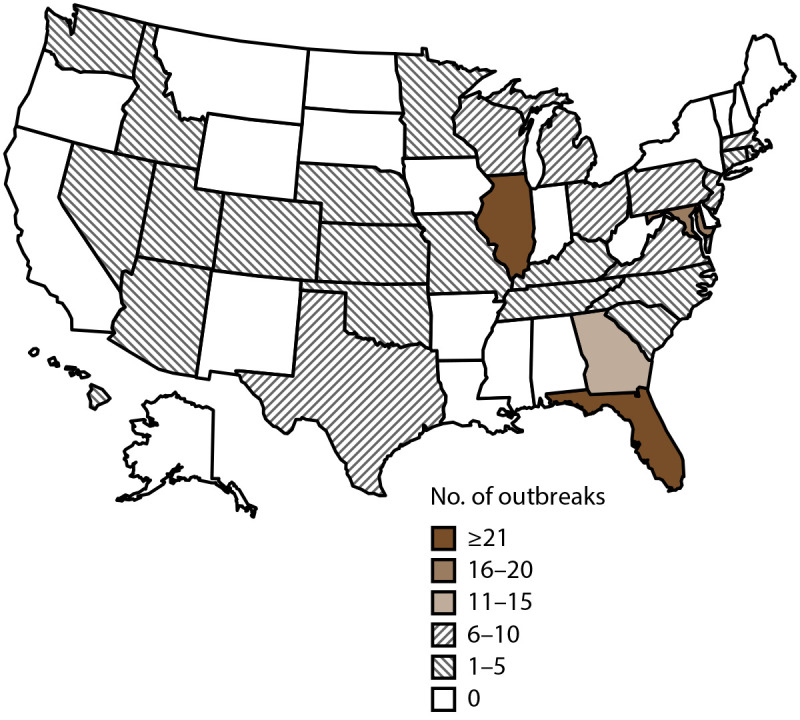
Number of reported drinking water-associated outbreaks,* by state of exposure — National Outbreak Reporting System, United States, 2015–2020 * N = 214 outbreaks.

**FIGURE 2:**
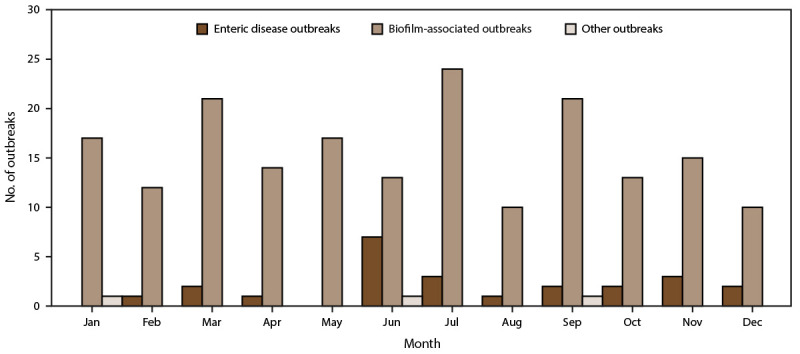
Number of reported drinking water-associated outbreaks,*^,†^ by month of earliest illness onset — National Outbreak Reporting System, United States, 2015–2020 * N = 214 outbreaks. ^†^ Other outbreaks refers to two outbreaks of unknown etiology and one outbreak caused by a chemical or toxin.

Public health officials from 28 states reported 214 outbreaks associated with drinking water during the surveillance period (Tables 1, 2, 3, 4, 5, and 6) (Figure 1). Reported outbreaks included 187 biofilm-associated, 24 enteric illness-associated, and three other (two unknown and one chemical or toxin) etiologies (Table 7) (Figure 2). Outbreaks resulted in at least 2,140 cases of illness, 563 hospitalizations (26% of cases), and 88 deaths (4% of cases). At least one etiologic agent was identified in 212 (99%) outbreaks (Tables 1, 2, 3, 4, 5, and 6).

**TABLE 1:** Waterborne disease outbreaks associated with drinking water,* by state and month of first case onset — Waterborne Disease and Outbreak Surveillance System, United States, 2015

State	Month	Etiology^†^	No. of cases	No. of hospitalizations	No. of deaths	Type of water system^§^	Water source description	Treatment	Treatment description	Water setting
Arizona	October	*Campylobacter jejuni* (S), norovirus (S), *Shigella sonnei* subgroup D (S)	250	0	0	Community	Well	No treatment	Not reported	Community or municipality
Florida	February	*Legionella* sp.	3	1	0	Community	Unknown	Disinfection	Unknown	Private residence
Florida	June	*L. pneumophila* serogroup 1	7	7	2	Community	Unknown	Disinfection	Chlorine dioxide	Assisted living or rehabilitation facility
Florida	September	*L. pneumophila* serogroup 1	2	2	0	Community	Well	Disinfection	Chloramine	Hotel, motel, lodge, or inn
Florida	November	*L. pneumophila*	5	4	0	Community	Unknown	Filtration – treatment plant	Unknown	Assisted living or rehabilitation facility
Florida	November	*L. pneumophila*	2	2	1	Community	Well	Disinfection	Chlorine	Hospital or health care facility
Florida	December	*L. pneumophila* serogroup 1	2	2	0	Community	Well	Disinfection	Not reported	Hotel, motel, lodge, or inn
Georgia	July	*L. pneumophila* serogroup 1	3	3	0	Community	Not reported	Disinfection	Unknown	Hospital or health care facility
Georgia	December	*L. pneumophila*	4	4	1	Community	Not reported	Disinfection	Chlorine	Hospital or health care facility
Georgia	December	*L. pneumophila* serogroup 1 (S)	6	6	0	Community	Not reported	Disinfection	Chlorine	Factory or industrial facility
Illinois	July	*L. pneumophila* serogroup 1	58	36	13	Individual or private	River or stream	Disinfection	Unknown	Long-term care facility
Illinois	October	*L. pneumophila* serogroup 1	2	1	0	Unknown	Not reported	Unknown	Not reported	Long-term care facility
Maryland	October	*L. pneumophila* serogroup 1	4	1	0	Community	Well	Unknown	Not reported	Hotel, motel, lodge, or inn
Michigan	May	*L. pneumophila* serogroup 1	43	43	5	Community	River or stream	Disinfection	Chlorine	Hospital or health care facility
Minnesota	December	*L. pneumophila* serogroup 1	2	2	0	Community	Unknown	No treatment	Not reported	Restaurant or cafeteria
Missouri	March	*L. pneumophila* serogroup 1	3	0	1	Not reported	Not reported	Not reported	Not reported	Not reported
New York	January	*L. pneumophila* serogroups 1 and 6	4	4	1	Community	Lake, reservoir, or impoundment	Disinfection	Chlorine, ultraviolet light	Hospital or health care facility
New York	March	*L. pneumophila* serogroups 1 and 4	5	3	1	Community	Lake, reservoir, or impoundment	Disinfection, other chemical	Chlorine, not reported	Hospital or health care facility
New York	March	Shiga-toxin producing *Escherichia coli*	6	0	0	Individual or private	Not reported	Unknown	Not reported	Farm or agricultural setting
New York	December	*Giardia duodenalis*	3	0	0	Individual or private	Well	No treatment	Not reported	Private residence
Ohio	January	Nontuberculosis mycobacteria (S)	4	0	0	Unknown	Not reported	Not reported	Not reported	Hospital or health care facility
Pennsylvania	July	*L. pneumophila*	2	2	0	Individual or private	Well	Disinfection	Chlorine	Casino
Utah	June	*Campylobacter jejuni*, *Giardia* (S)	9	1	0	Community^¶^	Well	No treatment, disinfection	Not reported, other	Private residence

**Abbreviation:** S = suspected.

* N = 23 outbreaks.

^†^ Etiologies listed are confirmed unless indicated as S. For multiple-etiology outbreaks, etiologies are listed in alphabetical order.

^§^ Community and noncommunity water systems are defined by the Environmental Protection Agency as public water systems that have ≥15 service connections or serve an average of ≥25 residents for ≥60 days per year. A community water system serves year-round residents of a community, subdivision, or mobile home park. A noncommunity water system serves an institution, industry, camp, park, hotel, or business and can be nontransient or transient. Nontransient systems serve ≥25 of the same persons for ≥6 months of the year but not year round (e.g., factories and schools), whereas transient systems provide water to places in which persons do not remain for long periods (e.g., restaurants, highway rest stations, and parks). Individual water systems are small systems not owned or operated by a water utility that have <15 connections or serve <25 persons.

^¶^ Two community water systems were reported for this outbreak.

**TABLE 2:** Waterborne disease outbreaks associated with drinking water,* by state and month of first case onset — Waterborne Disease and Outbreak Surveillance System, United States, 2016

State	Month	Etiology^†^	No. of cases	No. of hospitalizations	No. of deaths	Type of water system^§^	Water source description	Treatment	Treatment description	Water setting
Arizona	August	Norovirus GI.GI.3	32	0	0	Individual or private	Not reported	Unknown	Not reported	Hotel, motel, lodge, or inn
Colorado	September	*Legionella* sp.*, L. pneumophila* serogroup 1 (S)	2	2	0	Community	Lake, reservoir, or impoundment	Disinfection	Chlorine	Assisted living or rehabilitation facility
Florida	March	*L. pneumophila* serogroup 1 (S)	2	1	0	Individual or private	Well	Disinfection	Chlorine	Camp or cabin
Florida	March	*L. pneumophila* serogroup 1	4	2	0	Community	Unknown	Disinfection	Chlorine	Assisted living or rehabilitation facility
Florida	March	*L. pneumophila* serogroup 1	2	2	0	Community	Well	Disinfection	Chlorine	Hospital or health care facility
Florida	May	*L. pneumophila* serogroup 1	4	1	0	Community	Unknown	Disinfection	Chlorine	Assisted living or rehabilitation facility
Florida	June	*L. pneumophila* serogroup 1	6	6	0	Community	Unknown	Disinfection	Chlorine	Assisted living or rehabilitation facility
Florida	June	*L. pneumophila* serogroup 1	3	0	0	Community	Well	Disinfection	Unknown	Hotel, motel, lodge, or inn
Florida	September	*L. pneumophila* serogroup 1	2	2	0	Community	Well	Disinfection	Chloramine	Hotel, motel, lodge, or inn
Georgia	January	*L. pneumophila* serogroup 1	8	8	1	Community	Not reported	Disinfection	Chlorine	Hospital or health care facility
Georgia	May	*L. pneumophila* serogroup 1	2	2	0	Community	Not reported	Disinfection	Chlorine dioxide	Hospital or health care facility
Georgia	July	*L. pneumophila* serogroup 1 (S)	2	2	0	Community	Not reported	Disinfection	Chlorine	Hospital or health care facility
Georgia	October	*L. pneumophila, L. pneumophila* serogroup 1	2	1	0	Community	Not reported	Disinfection	Chlorine	Hotel, motel, lodge, or inn
Georgia	November	*L. pneumophila* serogroup 1	3	3	2	Community	Not reported	Disinfection	Chlorine	Hospital or health care facility
Hawaii	April	*Legionella, L. pneumophila* (S), *L. pneumophila* serogroup 1 (S)	2	2	0	Community	Unknown	Disinfection	Chlorine	Apartment or condominium
Illinois	February	*L. pneumophila* serogroup 1	5	2	0	Community	Not reported	Unknown	Not reported	Long-term care facility
Illinois	May	*L. pneumophila* serogroup 1	2	1	0	Community	Not reported	Disinfection	Chlorine	Casino
Illinois	July	*L. pneumophila* serogroup 1	6	2	0	Community	Not reported	Unknown	Not reported	Long-term care facility
Illinois	September	*L. pneumophila* serogroup 1	2	2	1	Community	Not reported	Unknown	Not reported	Hotel, motel, lodge, or inn
Maryland	March	*Pseudomonas aeruginosa*	8	8	2	Community	River or stream	Coagulation, flocculation, settling or sedimentation, filtration (treatment plant), disinfection, other chemical	Unknown, chlorine	Hospital or health care facility
Maryland	April	*L. pneumophila* serogroup 1	2	2	1	Community	Lake, reservoir, or impoundment	Coagulation, disinfection, flocculation, filtration (treatment plant), settling or sedimentation, other chemical	Chlorine, rapid sand	Other
Maryland	May	*L. pneumophila* serogroup 1	3	3	0	Community	Lake, reservoir, or impoundment	Coagulation, disinfection, flocculation, filtration (treatment plant), settling or sedimentation, other chemical	Chlorine, rapid sand	Other
Maryland	July	*L. pneumophila* serogroup 1	2	2	0	Community	River or stream	Coagulation, flocculation, settling or sedimentation, filtration (treatment plant), disinfection, other chemical	Unknown, chlorine	Hotel, motel, lodge, or inn
Maryland	July	*L. pneumophila* serogroup 1	3	1	0	Community	Lake, reservoir, or impoundment	Coagulation, disinfection, flocculation, filtration (treatment plant), settling or sedimentation, other chemical	Chlorine, rapid sand	Long-term care facility
Michigan	August	*L. pneumophila* serogroup 1	3	3	0	Community	River or stream	Disinfection	Chloramine	Hospital or health care facility
Missouri	June	*Giardia duodenalis* (S), Shiga-toxin producing *E. coli* O157:H7 (S), Shiga-toxin producing *Escherichia coli* O111 (S)	13	1	0	Community	Well	Disinfection	Chlorine	Camp or cabin
New York	October	*L. pneumophila* serogroup 1 (S)	4	2	0	Nontransient, noncommunity	Well	Disinfection, softening	Chlorine	Hospital or health care facility
Ohio	March	*L. pneumophila* serogroup 1	5	5	2	Community	Lake, reservoir, or impoundment	Disinfection	Chlorine	Apartment or condominium
Ohio	November	*L. pneumophila* serogroup 1	2	2	1	Community	Well	Disinfection	Chlorine	Long-term care facility
Oklahoma	October	Nontuberculosis mycobacteria	11	0	0	Community	Lake, reservoir, or impoundment	Unknown	Not reported	Unknown
Pennsylvania	February	*L. pneumophila* serogroup 1	4	3	1	Community	River or stream	Disinfection	Copper-silver ionization	Hospital or health care facility
Pennsylvania	May	*L. pneumophila* serogroup 1	3	3	1	Community	River or stream	Disinfection	Copper-silver ionization	Hospital or health care facility
Pennsylvania	May	*L. pneumophila* serogroup 1	6	3	1	Unknown	Not reported	Unknown	Not reported	Assisted living or rehabilitation facility
Pennsylvania	August	*L. pneumophila*	4	4	0	Community	River or stream	Unknown	Not reported	Other
Pennsylvania	October	*L. pneumophila* serogroup 1	3	2	0	Unknown	Not reported	Unknown	Not reported	Long-term care facility
South Carolina	November	*L. pneumophila* serogroup 1	4	3	0	Community	Unknown	Unknown	Not reported	Hotel, motel, lodge, or inn
Tennessee	July	*L. pneumophila* serogroup 1	42	24	0	Community	Unknown	Disinfection	Chlorine	Hotel, motel, lodge, or inn
Texas	December	*L. pneumophila* serogroup 1	4	4	1	Unknown	Not reported	Unknown	Not reported	Unknown
Virginia	October	Norovirus (S)	23	0	0	Individual or private	Well	No treatment	Not reported	Hall or meeting facility
Virginia	November	Norovirus GI	14	0	0	Individual or private	Well	No treatment	Not reported	Hall or meeting facility
Virginia	November	Norovirus GI.GI.3	31	1	0	Individual or private	Well	No treatment	Not reported	Hall or meeting facility
Virginia	November	Norovirus (S)	10	0	0	Individual or private	Well	No treatment	Not reported	Hall or meeting facility
Virginia	December	Norovirus GI.GI.3	25	0	0	Individual or private	Well	No treatment	Not reported	Hall or meeting facility
Washington	July	*L. pneumophila* serogroup 1	5	5	2	Community	Not reported	Unknown	Not reported	Hospital or health care facility

**Abbreviation:** S = suspected.

* N = 44 outbreaks.

^†^ Etiologies listed are confirmed unless indicated as S. For multiple-etiology outbreaks, etiologies are listed in alphabetical order.

^§^ Community and noncommunity water systems are defined by Environmental Protection Agency as public water systems that have ≥15 service connections or serve an average of ≥25 residents for ≥60 days per year. A community water system serves year-round residents of a community, subdivision, or mobile home park. A noncommunity water system serves an institution, industry, camp, park, hotel, or business and can be nontransient or transient. Nontransient systems serve ≥25 of the same persons for ≥6 months of the year but not year round (e.g., factories and schools), whereas transient systems provide water to places in which persons do not remain for long periods of time (e.g., restaurants, highway rest stations, and parks). Individual water systems are small systems not owned or operated by a water utility that have <15 connections or serve <25 persons.

**TABLE 3:** Waterborne disease outbreaks associated with drinking water,* by state and month of first case onset — Waterborne Disease and Outbreak Surveillance System, United States, 2017

State	Month	Etiology^†^	No. of cases	No. of hospitalizations	No. of deaths	Type of water system^§^	Water source description	Treatment	Treatment description	Water setting
Connecticut	March	*Legionella pneumophila* serogroup 1	3	1	0	Unknown	Not reported	Not reported	Not reported	Long-term care facility
Connecticut	March	*L. pneumophila* serogroup 1	2	2	1	Unknown	Not reported	Not reported	Not reported	Long-term care facility
Florida	January	*L. pneumophila* serogroup 1	3	2	0	Community	Unknown	Disinfection	Chlorine	Assisted living or rehabilitation facility
Florida	February	*L. pneumophila* serogroup 1	2	2	0	Community	Not reported	Disinfection	Unknown	Club (requires membership)
Florida	April	*L. pneumophila* serogroup 1	3	3	0	Community	Well	Disinfection	Chlorine	Community or municipality
Florida	May	*L. pneumophila* serogroup 1	2	2	0	Community	Not reported	Disinfection	Unknown	Club (requires membership)
Florida	May	*L. pneumophila* serogroup 1	2	2	0	Community	Not reported	Disinfection	Unknown	Hotel, motel, lodge, or inn
Florida	September	*L. pneumophila* serogroup 1 (S)	2	2	1	Community	Well	Disinfection	Chloramine	Hospital or health care facility
Florida	October	*L. pneumophila* serogroup 1	2	2	0	Other	Unknown	Disinfection	Chloramine	Resort
Florida	December	*L. pneumophila* serogroup 1	2	2	0	Other (hospital premise plumbing)	Not reported	Unknown	Not reported	Hospital or health care facility
Georgia	March	*L. pneumophila* serogroup 1	3	3	1	Community	Not reported	Disinfection	Unknown	Hospital or health care facility
Georgia	March	*L. pneumophila* serogroup 1	2	2	1	Community	Not reported	Disinfection	Unknown	Hospital or health care facility
Georgia	August	*L. pneumophila, L. pneumophila* serogroup 1	4	4	0	Community	Not reported	Disinfection	Chlorine	Hotel, motel, lodge, or inn
Illinois	March	*L. pneumophila* serogroup 1	6	4	1	Community	River or stream and well	Disinfection	Unknown	Long-term care facility
Illinois	May	*L. anisa, L. pneumophila* serogroup 1	2	2	1	Community	Lake, reservoir, or impoundment	Other chemical	Not reported	Hospital or health care facility
Maryland	July	*L. pneumophila* serogroups 1 and 5	7	6	1	Community	River or stream	Coagulation, flocculation, settling or sedimentation, filtration (treatment plant), disinfection, other chemical	Unknown, chlorine	Long-term care facility
Massachusetts	May	*Legionella* sp.	2	2	0	Community	Not reported	Unknown	Not reported	Hotel, motel, lodge, or inn
Michigan	June	*L. pneumophila* serogroup 1, *L. pneumophila* serogroups 2–14 (S)	4	4	0	Community	Other	Disinfection	Chlorine	Hospital or health care facility
Michigan	September	*L. pneumophila* serogroup 1 (S)	2	2	0	Community	Lake, reservoir, or impoundment	Disinfection	Chlorine	Hotel, motel, lodge, or inn
Minnesota	January	Copper	5	0	0	Community	River or Stream	Disinfection	Unknown	School, college, or university
Nebraska	February	*Campylobacter jejuni*	39	3	0	Community	Well	No treatment	Not reported	Unknown
Nevada	January	*L. pneumophila* serogroup 1	3	0	0	Community	Lake, reservoir or impoundment	Filtration (treatment plant)	Unknown	Hotel, motel, lodge, or inn
Nevada	January	*L. pneumophila* serogroup 1	2	0	0	Community	Lake, reservoir or impoundment	Filtration (treatment plant)	Unknown	Hotel, motel, lodge, or inn
Nevada	March	*L. pneumophila* serogroup 1	107	19	0	Community	Lake, reservoir or impoundment	Filtration (treatment plant)	Unknown	Hotel, motel, lodge, or inn
Nevada	June	*L. pneumophila* serogroup 1 (S)	2	0	0	Community	Lake, reservoir or impoundment	Filtration (treatment plant)	Unknown	Hotel, motel, lodge, or inn
Nevada	July	*L. pneumophila* serogroup 1	2	0	0	Community	Lake, reservoir or impoundment	Filtration (treatment plant)	Unknown	Hotel, motel, lodge, or inn
New York	March	*L. pneumophila* serogroup 1	2	2	0	Community	Lake, reservoir or impoundment	Coagulation, filtration (treatment plant)	Activated carbon, rapid sand	Hospital or health care facility
New York	July	*L. pneumophila* serogroup 1	4	4	0	Community	Lake, reservoir or impoundment	Disinfection, coagulation, filtration (treatment plant)	Chlorine, ultraviolet light	Hospital or health care facility
New York	October	*L. pneumophila* serogroup 1	2	2	0	Community	Well	Disinfection	Chlorine	Hospital or health care facility
North Carolina	March	*L. pneumophila* serogroup 1	4	4	0	Community	Not reported	Unknown	Not reported	Other
North Carolina	July	*L. pneumophila* serogroup 1	2	2	1	Community	Not reported	Unknown	Not reported	Long-term care facility
North Carolina	September	*L. pneumophila* serogroup 1	3	2	1	Community	Not reported	Unknown	Not reported	Long-term care facility
Ohio	May	*L. pneumophila* serogroup 1	5	5	1	Individual or private	Lake, reservoir, or impoundment	Disinfection	Chlorine	Hospital or health care facility
Ohio	July	*L. pneumophila* serogroup 1	2	2	0	Community	Lake, reservoir, or impoundment	Filtration (treatment plant)	Unknown	Long-term care facility
Pennsylvania	January	*L. pneumophila*	2	1	0	Community	Not reported	Aeration	Not reported	Hotel, motel, lodge, or inn
Wisconsin	April	*Legionella, L. pneumophila*, *L. pneumophila* serogroup 1 (S)	2	0	0	Transient, noncommunity	Well	No treatment	Not reported	Hotel, motel, lodge, or inn
Wisconsin	July	*L. pneumophila* serogroup 1	2	2	0	Community	Well	No treatment	Not reported	Long-term care facility
Wisconsin	September	*Giardia duodenalis*	6	0	0	Individual or private	Well	No treatment	Not reported	Private residence

**Abbreviation:** S = suspected.

* N = 38 outbreaks.

^†^ Etiologies are confirmed unless indicated as S.

^§^ Community and noncommunity water systems are defined by the Environmental Protection Agency as public water systems that have ≥15 service connections or serve an average of ≥25 residents for ≥60 days per year. A community water system serves year-round residents of a community, subdivision, or mobile home park. A noncommunity water system serves an institution, industry, camp, park, hotel, or business and can be nontransient or transient. Nontransient systems serve ≥25 of the same persons for ≥6 months of the year but not year round (e.g., factories and schools), whereas transient systems provide water to places in which persons do not remain for long periods of time (e.g., restaurants, highway rest stations, and parks). Individual water systems are small systems not owned or operated by a water utility that have <15 connections or serve <25 persons.

**TABLE 4:** Waterborne disease outbreaks associated with drinking water,* by state and month of first case onset — Waterborne Disease and Outbreak Surveillance System, United States, 2018

State	Month	Etiology^†^	No. of cases	No. of hospitalizations	No. of deaths	Type of water system^§^	Water source description	Treatment	Treatment description	Water setting
Florida	March	*Legionella pneumophila* serogroup 1	4	4	0	Community	Well	Disinfection	Chlorine	Long-term care facility
Florida	April	*Legionella* sp. (S)	3	3	1	Community	Other (aquifer)	Disinfection	Chlorine	Hospital or health care facility
Florida	April	Norovirus	21	0	0	Community	Well	Disinfection	Chlorine	Mobile home park
Florida	June	*L. pneumophila* serogroup 1	4	1	0	Community	Other	Disinfection	Chlorine	Hotel, motel, lodge, or inn
Florida	September	*L. pneumophila* serogroup 1	2	2	0	Community	Well	Disinfection	Chlorine	Hotel, motel, lodge, or inn
Florida	October	*L. pneumophila* serogroup 1	3	3	2	Community	Unknown	Disinfection	Chlorine	Long-term care facility
Florida	October	*L. pneumophila* (S)	2	2	0	Community	Other (aquifer)	Disinfection	Chlorine	Long-term care facility
Florida	November	*L. pneumophila* serogroup 1	3	2	0	Community	Not reported	Disinfection	Chlorine	Hotel, motel, lodge, or inn
Florida	November	*L. pneumophila* serogroup 1 (S)	2	2	1	Community	Other (aquifer)	Disinfection	Chlorine	Long-term care facility
Florida	December	*L. pneumophila* serogroup 1	2	2	0	Community	Not reported	Disinfection	Chlorine	Hotel, motel, lodge, or inn
Georgia	October	*L. pneumophila* serogroup 1	2	2	0	Community	Not reported	Disinfection	Chlorine	Hospital or health care facility
Idaho	July	*Campylobacter* sp.	24	0	0	Community	Other	Disinfection	Not reported	Subdivision or neighborhood
Illinois	February	*L. pneumophila* serogroup 1	4	2	0	Community	River or stream	Disinfection	Chlorine dioxide	Long-term care facility
Illinois	September	*L. pneumophila* serogroup 1	3	3	0	Community	Well	Disinfection	Chlorine	Other (grocery store)
Illinois	September	*L. pneumophila* serogroup 1 (S)	5	5	0	Community	Lake, reservoir, or impoundment	Disinfection	Chlorine	Hospital or health care facility
Illinois	November	*L. pneumophila* serogroup 1	2	2	0	Unknown	Not reported	Unknown	Not reported	Unknown
Illinois	November	*L. pneumophila* serogroup 1	3	2	1	Community	Well	Disinfection	Chlorine	Apartment or condominium
Illinois	December	*L. pneumophila* serogroup 1 (S)	2	2	0	Unknown	Not reported	Unknown	Not reported	Unknown
Illinois	December	*L. pneumophila* serogroup 1 (S)	2	2	0	Community	Lake, reservoir, or impoundment	Disinfection	Chlorine	Hospital or health care facility
Kansas	March	*L. pneumophila* (S)	2	0	0	Community	River or stream	Disinfection	Other	Hotel, motel, lodge, or inn
Maryland	January	*L. pneumophila* serogroup 1	2	2	0	Community	Lake, reservoir, or impoundment	Coagulation, disinfection, flocculation, filtration (treatment plant), settling or sedimentation, other chemical	Chlorine, rapid sand	Long-term care facility
Maryland	June	*L. pneumophila* serogroup 1	3	1	0	Community	Well	Unknown	Not reported	Apartment or condominium
Maryland	August	*L. pneumophila*	5	5	0	Community	Lake, reservoir, or impoundment	Coagulation, disinfection, flocculation, filtration (treatment plant), settling or sedimentation, other chemical	Chlorine, rapid sand	Apartment or condominium
Maryland	August	*L. pneumophila* (S)	2	2	0	Community	Lake, reservoir, or impoundment	Coagulation, disinfection, flocculation, filtration (treatment plant), settling or sedimentation, other chemical	Chlorine, rapid sand	Other, Other (shelter)
Maryland	September	*L. pneumophila* serogroup 1	3	1	0	Community	Well	Unknown	Not reported	Apartment or condominium
Maryland	September	*L. pneumophila*	3	3	0	Community	Lake, reservoir, or impoundment	Coagulation, disinfection, flocculation, filtration (treatment plant), settling or sedimentation, other chemical	Chlorine, rapid sand	Apartment or condominium
Maryland	September	*L. pneumophila* serogroup 1	3	1	1	Community	Well	Unknown	Not reported	Hotel, motel, lodge, or inn
Massachusetts	February	*Legionella*	2	2	0	Community	Not reported	Unknown	Not reported	Long-term care facility
Massachusetts	April	*L. pneumophila* serogroup 1	2	1	0	Unknown	Not reported	Unknown	Not reported	Long-term care facility
Massachusetts	September	*L. pneumophila* serogroup 1	2	2	0	Individual or private	Not reported	Unknown	Not reported	Private residence
Michigan	November	*L. pneumophila* serogroup 1	4	4	3	Community	River or stream	Disinfection	Chlorine	Hospital or health care facility
New York	July	*L. pneumophila* serogroup 1 (S)	2	0	0	Community	Lake, reservoir, or impoundment	Disinfection, coagulation, filtration (treatment plant), other (copper silver ionization)	Chlorine, ultraviolet light	Hospital or health care facility
Ohio	June	*L. pneumophila* serogroup 1	6	5	0	Community	Lake, reservoir, or impoundment	Coagulation	Not reported	Other
Rhode Island	July	*L. pneumophila* serogroup 1	2	1	1	Community	Lake, reservoir, or impoundment	Disinfection	Chlorine	Long-term care facility
Rhode Island	August	*L. pneumophila* serogroup 1	3	2	2	Community	Lake, reservoir, or impoundment	Disinfection	Chlorine	Long-term care facility
South Carolina	February	*L. pneumophila* serogroup 1	2	0	0	Community	Unknown	Disinfection	Unknown	Hotel, motel, lodge, or inn
Tennessee	June	*Cryptosporidium* sp., EAEC, EIEC, EPEC, *Giardia* sp., Norovirus (multiple genogroups)	693	3	0	Individual or private^¶^	Well	Filtration (home or point of use)	Activated carbon	Park (amusement)
Texas	June	*Shigella* sp.	10	1	0	Unknown	Not reported	Unknown	Not reported	Unknown
Texas	September	Not reported	3	1	0	Community or commercially bottled	Not reported	Not reported	Not reported	Not reported
Utah	April	*L. pneumophila* serogroup 1	3	0	1	Community	Not reported	Unknown	Not reported	Assisted living or rehabilitation facility
Wisconsin	January	*L. pneumophila* serogroup 1 (S)	2	2	2	Community	Lake, reservoir, or impoundment	Disinfection	Chlorine	Long-term care facility
Wisconsin	June	*L. pneumophila* serogroup 1	2	2	1	Community	Unknown	No treatment	Not reported	Hotel, motel, lodge, or inn
Wisconsin	July	*L. pneumophila* serogroup 1	2	2	0	Community	Well	Disinfection	Chlorine	Long-term care facility
Wisconsin	November	*L. pneumophila* serogroup 1	14	12	2	Community	Unknown	Disinfection	Chlorine	Hospital or health care facility

**Abbreviations:** EAEC = enteroaggregative *Escherichia coli*; EIEC = enteroinvasive *Escherichia coli*; EPEC = enteropathogenic *Escherichia coli*; S = suspected.

* N = 44 outbreaks.

^†^ Etiologies listed are confirmed, unless indicated as S. For multiple-etiology outbreaks, etiologies are listed in alphabetical order.

^§^ Community and noncommunity water systems are defined by the Environmental Protection Agency as public water systems that have ≥15 service connections or serve an average of ≥25 residents for ≥60 days per year. A community water system serves year-round residents of a community, subdivision, or mobile home park. A noncommunity water system serves an institution, industry, camp, park, hotel, or business and can be nontransient or transient. Nontransient systems serve ≥25 of the same persons for ≥6 months of the year but not year round (e.g., factories and schools), whereas transient systems provide water to places in which persons do not remain for long periods of time (e.g., restaurants, highway rest stations, and parks). Individual water systems are small systems not owned or operated by a water utility that have <15 connections or serve <25 persons.

^¶^ Water system classified by the regulatory authority as a noncommunity water system because of the outbreak investigation.

**TABLE 5:** Waterborne disease outbreaks associated with drinking water,* by state and month of first case onset — Waterborne Disease and Outbreak Surveillance System, United States, 2019

State	Month	Etiology^†^	No. of cases	No. of hospitalizations	No. of deaths	Type of water system^§^	Water source description	Treatment	Treatment description	Water setting
Florida	January	*Legionella pneumophila, L. pneumophila* serogroup 1	3	3	0	Community	Other (aquifer)	Disinfection	Chlorine	Hospital or health care facility
Florida	January	*L. pneumophila*	3	3	0	Community	Other (aquifer)	Disinfection	Chlorine	Assisted living or rehabilitation facility
Florida	February	*L. pneumophila* serogroup 1	2	2	0	Community	Other (aquifer)	Disinfection	Chlorine	Long-term care facility
Florida	May	*Legionella* (S), *L. pneumophila* serogroup 1	2	1	0	Community	Unknown	Disinfection	Unknown	Long-term care facility
Florida	June	*L. pneumophila*	2	1	0	Community	Other (aquifer)	Disinfection	Chlorine	Long-term care facility
Florida	June	*Legionella* sp.	4	4	2	Community	Unknown	Disinfection	Chloramine	Long-term care facility
Florida	July	*L. pneumophila* serogroup 1	3	3	1	Community	Unknown	Disinfection	Chlorine	Hospital or health care facility
Florida	August	*L. pneumophila, L. pneumophila* serogroup 1 (S)	2	2	0	Community	Other (aquifer)	Disinfection	Chlorine	Apartment or condominium
Florida	August	*L. pneumophila* serogroup 1 (S)	2	2	0	Community	Well	Disinfection	Chloramine	Assisted living or rehabilitation facility
Florida	September	*L. pneumophila* serogroup 1	5	5	0	Community	Other (aquifer)	Disinfection	Chlorine	Hospital or health care facility
Florida	September	*L. pneumophila*	2	2	0	Community	Other (aquifer)	Disinfection	Chlorine	Long-term care facility
Florida	November	*L. pneumophila* serogroup 1	2	1	0	Community	Well	Disinfection	Chloramine	Private residence
Florida	November	*L. pneumophila* serogroup 1	2	0	0	Community	Not reported	Disinfection	Chlorine	Hotel, motel, lodge, or inn
Georgia	February	*L. pneumophila* serogroup 1	2	2	0	Community	Not reported	Unknown	Not reported	Hospital or health care facility
Illinois	January	*L. pneumophila* serogroup 1	2	2	1	Community	River or stream	Disinfection	Chloramine	Other (veterans home)
Illinois	February	*L. pneumophila* serogroups 1 and 6	3	3	2	Community	Lake, reservoir, or impoundment	Disinfection	Chlorine	Hospital or health care facility
Illinois	February	*L. pneumophila* serogroup 1	2	2	1	Community	Well	Disinfection	Chlorine	Long-term care facility
Illinois	May	*L. pneumophila* serogroup 1	2	2	1	Community	Lake, reservoir, or impoundment	Disinfection	Chlorine	Hospital or health care facility
Illinois	May	*L. pneumophila* serogroup 1	2	2	0	Community	Lake, reservoir, or impoundment	Disinfection	Chlorine	Hospital or health care facility
Illinois	May	*L. pneumophila* serogroup 1	7	7	1	Community	Lake, reservoir, or impoundment	Disinfection	Chlorine	Hospital or health care facility
Illinois	May	*L. pneumophila* serogroup 1	3	2	2	Community	Lake, reservoir, or impoundment	Disinfection	Chlorine	Apartment or condominium
Illinois	June	*L. pneumophila* serogroup 1	2	2	0	Community	Lake, reservoir, or impoundment	Disinfection	Chlorine	Hotel, motel, lodge, or inn
Illinois	July	*L. pneumophila* serogroup 1 (S)	2	1	0	Community	Lake, reservoir, or impoundment	Disinfection	Chlorine	Hotel, motel, lodge, or inn
Illinois	October	*L. pneumophila* serogroup 1	2	2	0	Community	River or stream	Disinfection	Chloramine	Hospital or health care facility
Illinois	December	*L. pneumophila* serogroup 1	2	1	0	Community	Lake, reservoir, or impoundment	Disinfection	Chlorine	Long-term care facility
Kentucky	November	*L. pneumophila* (S)	8	1	1	Community	Not reported	Unknown	Not reported	Long-term care facility
Maryland	July	*L. pneumophila* serogroup 1	2	1	0	Community	Well	Unknown	Not reported	Apartment or condominium
Massachusetts	March	*L. pneumophila* serogroup 1 (S)	2	2	0	Unknown	Not reported	Unknown	Not reported	Long-term care facility
Massachusetts	September	*L. pneumophila* serogroup 1, *L. pneumophila* serogroups other than 1–6	4	3	0	Unknown	Not reported	Unknown	Not reported	Hospital or health care facility
Massachusetts	September	*L. pneumophila* serogroup 1 (S)	2	2	0	Unknown	Not reported	Unknown	Not reported	Hospital or health care facility
Massachusetts	September	*L. pneumophila* serogroup 1	9	3	0	Community	Unknown	No treatment	Not reported	Apartment or condominium
Michigan	April	*L. pneumophila* serogroup 1	2	2	0	Community	Lake, reservoir, or impoundment	Disinfection	Chlorine	Factory or industrial facility
Michigan	June	*L. pneumophila* serogroup 1 (S)	2	2	1	Community	Lake, reservoir, or impoundment	Disinfection	Chlorine	Hospital or health care facility
Ohio	June	Norovirus GII.GII.3[P12], Shiga-toxin producing *Escherichia coli* O103	30	0	0	Individual or private	Not reported	No treatment	Not reported	Hall or meeting facility
Pennsylvania	April	*L. pneumophila* serogroup 1 (S)	5	5	0	Community	Unknown	Unknown	Not reported	Hospital or health care facility
Pennsylvania	July	*L. pneumophila* serogroup 1 (S)	2	2	0	Nontransient, noncommunity	Unknown	Unknown	Not reported	Hospital or health care facility
Texas	June	*Shigella* sp.	7	0	0	Unknown	Not reported	Unknown	Not reported	Unknown
Texas	June	*Cryptosporidium* sp.	3	0	0	Unknown	Not reported	Unknown	Not reported	Unknown
Texas	July	*Shigella* sp.	3	0	0	Unknown	Not reported	Unknown	Not reported	Unknown
Texas	September	*Shigella* sp. (S)	4	0	0	Unknown	Not reported	Unknown	Not reported	Unknown
Virginia	July	*C.hominis*	41	0	0	Community	Not reported	Disinfection	Chlorine	Military facility
Virginia	July	*L. pneumophila*	2	2	0	Unknown	Not reported	Unknown	Not reported	Long-term care facility
Virginia	November	*L. pneumophila*	3	0	0	Unknown	Not reported	Unknown	Not reported	Hospital or health care facility
Wisconsin	January	*L. pneumophila* serogroup 1 (S)	2	2	1	Community	Lake, reservoir, or impoundment	Disinfection	Ozone	Assisted living or rehabilitation facility
Wisconsin	October	*L. pneumophila* serogroup 1 (S)	2	2	0	Individual or private	Well	No Treatment	Not reported	Hotel, motel, lodge, or inn

**Abbreviation:** S = suspected.

* N = 45 outbreaks.

^†^ Etiologies listed are confirmed, indicated as S. For multiple-etiology outbreaks, etiologies are listed in alphabetical order.

^§^ Community and noncommunity water systems are defined by the Environmental Protection Agency as public water systems that have ≥15 service connections or serve an average of ≥25 residents for ≥60 days per year. A community water system serves year-round residents of a community, subdivision, or mobile home park. A noncommunity water system serves an institution, industry, camp, park, hotel, or business and can be nontransient or transient. Nontransient systems serve ≥25 of the same persons for ≥6 months of the year but not year round (e.g., factories and schools), whereas transient systems provide water to places in which persons do not remain for long periods of time (e.g., restaurants, highway rest stations, and parks). Individual water systems are small systems not owned or operated by a water utility that have <15 connections or serve <25 persons.

**TABLE 6:** Waterborne disease outbreaks associated with drinking water,* by state and month of first case onset — Waterborne Disease and Outbreak Surveillance System, United States, 2020

State	Month	Etiology^†^	No. of cases	No. of hospitalizations	No. of deaths	Type of water system^§^	Water source description	Treatment	Treatment description	Water setting
Florida	January	*Legionella* sp. (S)	2	2	0	Community	Unknown	Disinfection	Unknown	Hospital or health care facility
Florida	January	*Legionella* sp. (S)	2	2	1	Community	Unknown	Disinfection	Chlorine	Long-term care facility
Florida	January	*L. pneumophila* serogroup 1	2	2	0	Community	Not reported	Disinfection	Chlorine	Hospital or health care facility
Florida	March	*Legionella* sp. (S)	2	2	0	Community	Unknown	Disinfection	Unknown	Long-term care facility
Florida	March	*Legionella* sp.	2	2	1	Community	Unknown	Disinfection	Chloramine	Assisted living or rehabilitation facility
Florida	March	*Legionella* sp.	2	2	0	Community	Other (aquifer)	Disinfection	Chlorine	Hospital or health care facility
Florida	April	*L. pneumophila* (S)	2	2	0	Community	Not reported	Disinfection	Chlorine	Long-term care facility
Florida	April	*L. pneumophila*	2	2	1	Community	Other (aquifer)	Disinfection	Chlorine	Hospital or health care facility
Florida	April	*Legionella* sp. (S)	2	2	0	Community	Other (aquifer)	Disinfection	Unknown	Assisted living or rehabilitation facility
Florida	September	*L. pneumophila* serogroup 1 (S)	2	2	1	Community	Other (aquifer)	Disinfection	Chlorine	Hospital or health care facility
Hawaii	March	*Giardia* sp. (S)	2	0	0	Community	Unknown	Unknown	Not reported	Unknown
Illinois	January	*L. pneumophila* serogroup 1	2	2	0	Community	Lake, reservoir, or impoundment	Disinfection	Chlorine	Long-term care facility
Illinois	August	*L. anisa* (S), *L. pneumophila* serogroup 1 (S)	3	3	1	Community	Unknown	Disinfection	Chlorine	Apartment or condominium
Illinois	September	*L. pneumophila* serogroup 1	2	2	0	Community	River or stream	Disinfection	Chloramine	Prison or jail (juvenile or adult)
Maryland	July	*L. pneumophila* serogroup 1	2	1	0	Community	Well	Unknown	Not reported	Hotel, motel, lodge, or inn
Massachusetts	June	Unknown	24	0	0	Community	Not reported	Unknown	Not reported	Private residence
Massachusetts	August	*L. pneumophila* serogroup 1	3	3	0	Unknown	Not reported	Unknown	Not reported	Hospital or health care facility
Ohio	February	*L. pneumophila* (S)	2	2	0	Community	Other (city)	Unknown	Not reported	Hospital or health care facility
Pennsylvania	April	*L. pneumophila* serogroup 1 (S)	3	3	0	Community	Not reported	Unknown	Not reported	Apartment or condominium
Wisconsin	April	*L. pneumophila* serogroup 1 (S)	2	2	0	Community	Unknown	No treatment	Not reported	Long-term care facility

**Abbreviation:** S = suspected.

* N = 20 outbreaks.

^†^ Etiologies listed are confirmed unless indicated as S.

^§^ Community and noncommunity water systems are defined by the Environmental Protection Agency as public water systems that have ≥15 service connections or serve an average of ≥25 residents for ≥60 days per year. A community water system serves year-round residents of a community, subdivision, or mobile home park. A noncommunity water system serves an institution, industry, camp, park, hotel, or business and can be nontransient or transient. Nontransient systems serve ≥25 of the same persons for ≥6 months of the year but not year round (e.g., factories and schools), whereas transient systems provide water to places in which persons do not remain for long periods of time (e.g., restaurants, highway rest stations, and parks). Individual water systems are small systems not owned or operated by a water utility that have <15 connections or serve <25 persons.

**TABLE 7:** Rank order (most common to least common) of etiology, water system, water source, and contributing factor types for drinking water-associated outbreaks and associated cases of illness — United States, 2015–2020

Characteristic or rank	Outbreaks*		Cases*	
	Category	No. (%)	Category	No. (%)
**Etiology**				
1	Bacterium (biofilm associated^†^), *Legionella*	184 (86.0)	Multiple (enteric)^§^	995 (46.5)
2	Virus	7 (3.3)	Bacterium (biofilm associated), *Legionella*	786 (36.7)
3	Bacterium (enteric)	7 (3.3)	Virus (enteric)	156 (7.3)
4	Multiple (enteric)	5 (2.3)	Bacterium (enteric)	93 (4.3)
5	Parasite (enteric)	5 (2.3)	Parasite (enteric)	55 (2.6)
6	Bacterium (biofilm associated), non-*Legionella*	3 (1.4)	Unknown	27 (1.3)
7	Unknown	2 (0.9)	Bacterium (biofilm associated), non-*Legionella*	23 (1.1)
8	Chemical or toxin	1 (0.5)	Chemical or toxin	5 (0.2)
**Water system**				
1	Community	169 (79.0)	Community	1,106 (51.7)
2	Unknown	22 (10.3)	Individual or private	944 (44.1)
3	Individual or private	17 (7.9)	Unknown	75 (3.5)
4	Noncommunity	3 (1.4)	Noncommunity	8 (0.4)
5	Other	2 (0.9)	Other	4 (0.2)
6	Not reported	1 (0.5)	Not reported	3 (0.1)
**Water source**				
1	Groundwater	82 (38.3)	Groundwater	1,348 (63.0)
2	Unknown	61 (28.5)	Surface water	384 (17.9)
3	Surface water	57 (26.6)	Unknown	309 (14.4)
4	Mixed	11 (5.1)	Mixed	89 (4.2)
5	Not reported	3 (1.4)	Not reported	10 (0.5)
**Contributing factor type**				
1	Premise point of use	85 (39.7)	Multiple	1,374 (64.2)
2	Not reported	70 (32.7)	Premise point of use	389 (18.2)
3	Multiple	46 (21.5)	Not reported	238 (11.1)
4	Source	7 (3.3)	Source	124 (5.8)
5	Distribution	6 (2.8)	Distribution	15 (0.7)

* N = 214 outbreaks; N = 2,140 cases.

^†^ Biofilm-associated drinking water outbreaks in this analysis include outbreaks caused by *Legionella* (n = 184), nontuberculous *Mycobacteria* (n = 2), and *Pseudomonas* (n = 1).

^§^ Multiple-etiology outbreaks include two enteric bacterial and parasitic; two enteric bacterial and viral; and one enteric bacterial, parasitic, or viral etiologic category.

#### Water Systems, Sources, and Contributing Factors

Community or noncommunity water systems (i.e., public) were linked with 172 (80%) outbreaks, 22 (10%) outbreaks with unknown water systems, 17 (8%) with individual or private systems (i.e., unregulated), and two (0.9%) with other systems; one system type (0.5%) was not reported. Water from individual or private water systems was implicated in 944 (44%) cases, 52 (9%) hospitalizations, and 14 (16%) deaths (Tables 1–6). Drinking water systems with groundwater sources accounted for 82 (38%) outbreaks, surface water sources accounted for 57 (27%) outbreaks, and unknown water sources accounted for 61 (29%) outbreaks (Table 7). A total of 454 contributing factors (practices and factors that led to the outbreak) were reported for 144 (67%) outbreaks (Tables 8 and 9). A total of 393 contributing factors were reported for biofilm-associated outbreaks and 61 for enteric illness-associated outbreaks.

**TABLE 8:** Rank order (most common to least common) of contributing factors reported for *Legionella* and other biofilm-associated* drinking water outbreaks^†^ — United States, 2015–2020

Type or rank	Contributing factor^§^	No. (%)^¶^
**Source**		
1	Unknown	10 (2.5)
2	None	2 (0.5)
3	Use of an alternate source of water by a water utility	1 (0.3)
4	Underchlorinated	1 (0.3)
5	Other	1 (0.3)
**Treatment**		
1	Unknown	8 (2.0)
2	Construction or repair of pipes or components without evidence of contamination	5 (1.3)
3	No disinfection	4 (1.0)
4	Chronically inadequate disinfection	2 (0.5)
5	Contamination during construction or repair of pipes or components	2 (0.5)
6	Change in treatment process	2 (0.5)
7	Temporary interruption of disinfection	1 (0.3)
8	Deficiencies in other treatment processes	1 (0.3)
9	Pipe or component failure or break (e.g., pipes, tanks, or valves)	1 (0.3)
**Distribution**		
1	*Legionella-*promoting water temperatures and chlorine levels within the potable water system inside building**	15 (3.8)
2	Water temperature ≥30°C (≥86°F)	8 (2.0)
3	Unknown	7 (1.8)
4	Aging water distribution components (e.g., pipes, tanks, or valves)	5 (1.3)
5	Amplification of *Legionella pneumophila* in colonized municipal water taking place inside hotel**	4 (1.0)
6	Low water pressure or change in water pressure in distribution system	4 (1.0)
7	Lowest hot water temperature documents from 106°F (41.1°C) to 109.5°F (43.1°C) suspected**	4 (1.0)
8	Stagnation of water because of sporadic occupancy**	4 (1.0)
9	Construction or repair of mains without evidence of contamination	3 (0.8)
10	Multiple dead legs (i.e., piping subject to low or no flow because of design or decreased water use) in facility before remediation (i.e., after outbreak)**	2 (0.5)
11	Contamination of mains during construction or repair	2 (0.5)
12	Contamination of storage facility	2 (0.5)
13	Pipe or component failure or break (e.g., pipes, tanks, or valves)	2 (0.5)
14	Cross-connection of potable and nonpotable water pipes resulting in backflow	1 (0.3)
15	Mixing of treated water from different sources	1 (0.3)
16	Corrosion in or leaching from pipes or storage tanks	1 (0.3)
**Premise or point of use**		
1	*Legionella* species in water system	67 (17.0)
2	*Legionella-*promoting water temperatures and chlorine levels within potable water system inside building	35 (8.9)
3	Water temperature ≥30°C (≥86°F)	28 (7.1)
4	Aging plumbing components (e.g., pipes, tanks, or valves)	27 (6.9)
5	Deficiency in building or home-specific water treatment after water meter or property line	15 (3.8)
6	Contamination at point of use (tap)	13 (3.3)
7	*Legionella-*promoting chlorine levels within potable water system inside building	11 (2.8)
8	Temperature control in hot water systems	10 (2.5)
9	Deficiency or contamination of equipment or devices using or distributing water	9 (2.3)
10	Contamination of plumbing during construction or repair	9 (2.3)
11	Stagnation of water because of sporadic occupancy	9 (2.3)
12	Construction or repair of plumbing without evidence of contamination	7 (1.8)
13	Dead-end water lines (i.e., piping subject to low or no flow because of design or decreased water use)	6 (1.5)
14	Unknown	6 (1.5)
15	Low pressure or change in water pressure in plumbing	5 (1.3)
16	Pipe or component failure or break (e.g., pipes, tanks, or valves)	4 (1.0)
17	Hot water temperature >115°F (46.1°C) at point of use	4 (1.0)
18	Amplification of *Legionella pneumophila* from colonized municipal water in hotel	3 (0.8)
19	Lack of backflow prevention in plumbing	3 (0.8)
20	Lack of adequate flushing following construction	3 (0.8)
21	Hurricane	2 (0.5)
22	Corrosion in or leaching from pipes or storage tanks	2 (0.5)
23	Other	2 (0.5)
24	High- and low-pressure humidification devices	1 (0.3)
25	Contamination at point of use (unknown)	1 (0.3)
26	Improper use of disinfection	1 (0.3)
27	Multiple dead legs (i.e., piping subject to low or no flow because of design or decreased water use) identified in facility	1 (0.3)
28	No supplemental disinfection in facility	1 (0.3)
29	Expansion of plumbing system in phases upon existing infrastructure	1 (0.3)
30	Cross-connection of potable and nonpotable water pipes resulting in backflow	1 (0.3)
**Total contributing factors**		**393 (100.0)**

* Biofilm-associated drinking water outbreaks in this analysis include outbreaks caused by *Legionella* (n = 184), nontuberculous *Mycobacteria* (n = 2), and *Pseudomonas* (n = 1).

^†^ N = 187 outbreaks. Outbreaks include those involving community (n = 158), unknown (n = 17), individual or private (n = 6), noncommunity (n = 3), and other (n = 2) water systems.

^§^ One outbreak might have multiple contributing factors reported.

^¶^ Percentage is calculated using a denominator of 393 because 393 is the total number of contributing factors reported for the 187 outbreaks.

** Reported as distribution factor types by users but aligns more closely with premise or point-of-use factor types.

**TABLE 9:** Rank order (most common to least common) of 61 contributing factors reported for outbreaks of enteric illness associated* with drinking water — United States, 2015–2020

Type or rank	Contributing factor^§^	No. (%)^¶^
**Source**		
1	Contamination through limestone or fissured rock (e.g., karst)	7 (11.5)
2	Improper construction or location of a well or spring	7 (11.5)
3	Flooding or heavy rains	5 (8.2)
4	Unknown	2 (3.3)
5	Domestic animal contamination (e.g., livestock, concentrated feeding operations, or pets)	2 (3.3)
6	Groundwater under direct influence of surface water (e.g., shallow well)	2 (3.3)
7	Underchlorinated	1 (1.6)
8	Malfunctioning onsite wastewater treatment system	1 (1.6)
9	Irrigation water was cross-connected with drinking water in neighborhood	1 (1.6)
10	Combined sewer overflow	1 (1.6)
11	Pump failure	1 (1.6)
12	Well cap under influence of surface water	1 (1.6)
13	Low water table (e.g., drought or overpumping)	1 (1.6)
14	Fill hose was in direct contact with the ground	1 (1.6)
15	Water system intake failure (e.g., cracked well casing or cracked intake pipe)	1 (1.6)
**Treatment**		
1	No disinfection	9 (14.8)
2	No filtration	5 (8.2)
3	Chronically inadequate disinfection	2 (3.3)
4	Pipe or component failure or break (e.g., pipes, tanks, or valves)	1 (1.6)
5	Inadequate filtration	1 (1.6)
6	Unknown	1 (1.6)
**Distribution**		
1	Cross-connection of potable and nonpotable water pipes resulting in backflow	1 (1.6)
2	Low pressure or change in water pressure in the distribution system	1 (1.6)
3	Unknown	1 (1.6)
**Premise or point of use**		
1	Unknown	2 (3.3)
2	Pump failure	1 (1.6)
3	Cross-connection of potable and nonpotable water pipes resulting in backflow	1 (1.6)
4	Lack of adequate flushing following construction	1 (1.6)
**Total**		**61 (100.0)**

* Enteric illness-associated drinking water outbreaks include outbreaks caused by *Campylobacter* (n = 2), *Cryptosporidium* (n = 2), *Escherichia coli* (n = 1), *Giardia* (n = 3), norovirus (n = 7), *Shigella* (n = 4), and multiple etiologies (n = 5).

^†^ N = 24 outbreaks. Outbreaks include those involving individual or private (n = 11), community (n = 8), and unknown (n = 5) water systems.

^§^ One outbreak might have multiple contributing factors reported.

^¶^ Percentage is calculated using a denominator of 61 because 61 is the total number of contributing factors reported for the 24 outbreaks.

### Enteric Illness-Associated Etiologies

Outbreaks of enteric illness included 24 (11%) reports implicating *Campylobacter* (n = 2; 1%), *Cryptosporidium* (n = 2; 1%), *Escherichia coli* (n = 1; 0.5%), *Giardia* (n = 3; 1%), norovirus (n = 7; 3%), *Shigella* (n = 4; 2%), and multiple etiologies (n = 5; 2%) (Tables 1, 2, 3, 4, 5, and 6). The enteric illness outbreaks resulted in 1,299 (61%) cases, 10 (2%) hospitalizations, and no deaths. Seventeen outbreaks were linked to norovirus, *Shigella*, *Campylobacter*, or multiple etiology outbreaks including these three pathogens and were implicated in 1,225 (57%) cases.

#### Water System and Water Source

The largest number of cases reported for a single outbreak was 693 (32%). This outbreak was linked to water from an individual or private water system that was contaminated with norovirus and enteropathogenic *E. coli* (Table 4). When water source (e.g., groundwater) was known (n = 14), wells were identified in 13 (93%) of enteric illness outbreaks, regardless of water system (Tables 1, 2, 3, 4, 5, and 6).

#### Contributing Factors

A total of 61 (13%) contributing factors were reported for enteric illness outbreaks (Table 9). Water source was the most cited contributing factor type for enteric illness outbreaks, described by 34 (56%) individual contributing factors. Contamination through limestone or fissured rock (e.g., karst) (n = 7; 11%), improper construction or location of a well or spring (n = 7; 11%), and flooding or heavy rains (n = 5; 8%) were the most reported source water contributing factors for enteric illness outbreaks. No disinfection (n = 9, 15%), no filtration (n = 5, 8%), and chronically inadequate disinfection (n = 2; 3%) were the most frequently reported treatment contributing factors for enteric disease outbreaks (Table 9).

### Biofilm-Associated Etiologies

Biofilm-associated outbreaks comprised 184 *Legionella* (86%), two NTM (1%), and one *Pseudomonas* (0.5%) outbreaks (Table 7). *Legionella*-associated outbreaks generally increased in number over the study period (14 in 2015, 31 in 2016, 30 in 2017, 34 in 2018, 33 in 2019, and 18 in 2020). The *Legionella* outbreaks resulted in 786 (37%) cases (Table 7), 544 (97%) hospitalizations, and 86 (98%) deaths (Tables 1, 2, 3, 4, 5, and 6).

#### Water System

*Legionella* was the most implicated etiology in public water system outbreaks, associated with 160 (92%) outbreaks, 666 (60%) cases, 462 (97%) hospitalizations, and 68 (97%) deaths related to community and noncommunity water systems (Tables 1, 2, 3, 4, 5, and 6). Individual or private water system outbreaks associated with *Legionella* resulted in 71 (8%) cases, 48 (92%) hospitalizations, and 14 (100%) deaths.

#### Contributing Factors

A total of 393 (87%) contributing factors were reported for *Legionella* and other biofilm pathogen-associated outbreaks (Table 8). Premise or point of use was the most cited contributing factor type for all biofilm-associated pathogen outbreaks and was linked with 287 (73%) individual contributing factors. The most reported premise or point of use contributing factors were *Legionella* species in water system (n = 67; 17%), *Legionella* growth-promoting water temperatures and permissive chlorine levels within the building potable water system (n = 35; 9%), water temperature ≥86°F (≥30°C) (n = 28; 7%), and aging plumbing components (e.g., pipes, tanks, and valves) (n = 27; 7%) (Table 8).

### Water Treatment and Water Treatment Methods

A total of 183 (86%) outbreak reports contained information about water treatment. Among all outbreaks, disinfection was the reported water treatment for 116 (54%) drinking water systems, unknown water treatment for 49 (23%) drinking water systems, and no water treatment for 17 (8%) drinking water systems (Tables 1, 2, 3, 4, 5, and 6). Seventy-nine outbreak reports (37%) indicated that chlorine was the water treatment method (e.g., description), 99 (46%) reported unknown or no treatment description, and 12 (6%) reported chloramine as the treatment description.

### Settings

**FIGURE 3:**
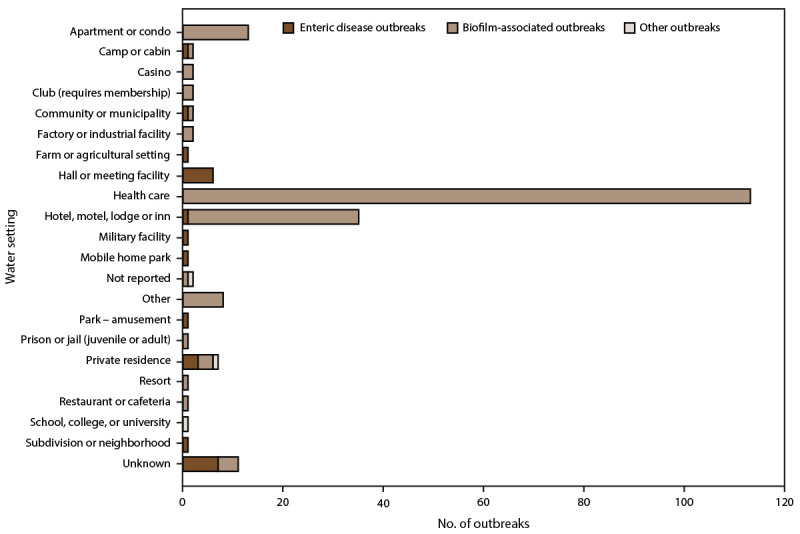
Number of reported drinking water-associated outbreaks,* by water setting of exposure^†,§^ — National Outbreak Reporting System, United States, 2015–2020^¶^ * N = 214 outbreaks. ^†^ Health care setting includes assisted living or rehabilitation facilities, hospital or health care facilities, and long-term care facilities. ^§^ Other setting includes grocery store, veterans’ home, shelter, and other (not specified). ^¶^ Other outbreaks refers to two outbreaks of unknown etiology and one outbreak caused by a chemical or toxin.

Hospital or health care facility, long-term care facility, and assisted living or rehabilitation facility (i.e., health care) were identified as the exposure settings in 113 (53%) outbreaks, 456 (21%) cases, 372 (66%) hospitalizations, and 75 (87%) deaths (Figure 3). Furthermore, in the health care facility setting, *Legionella* was implicated in 111 (52%) outbreaks, 444 (21%) cases, 364 (65%) hospitalizations, and 73 (85%) deaths. Hotels, motels, lodges, or inns were implicated in 35 (16%) outbreaks, 225 (11%) cases, 85 (15%) hospitalizations, and three (3%) deaths, all of which were caused by *Legionella* (Tables 1, 2, 3, 4, 5, and 6). Finally, *Legionella* was reported to NORS in 2015 and 2019 as the cause of three outbreaks in private residences resulting in seven (0.3%) cases, four (0.7%) hospitalizations, and no deaths (Tables 1 and 4) (Figure 3) (*2*).

## Discussion

**FIGURE 4:**
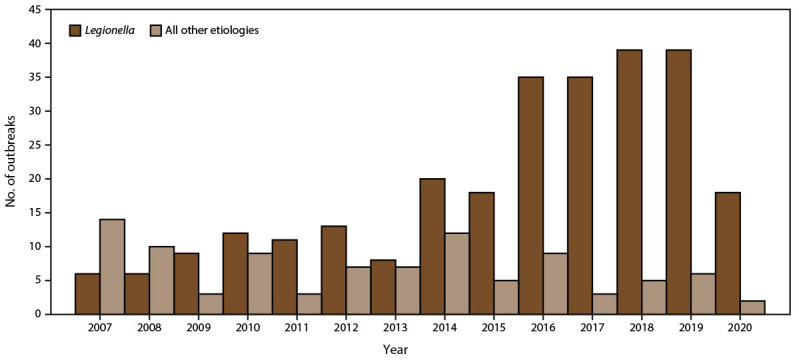
Number of reported drinking water-associated outbreak etiologies,* by *Legionella* compared with all other etiologies — Waterborne Disease and Outbreak Surveillance System, United States, 2007–2020 * N = 366 outbreak etiologies.

**FIGURE 5:**
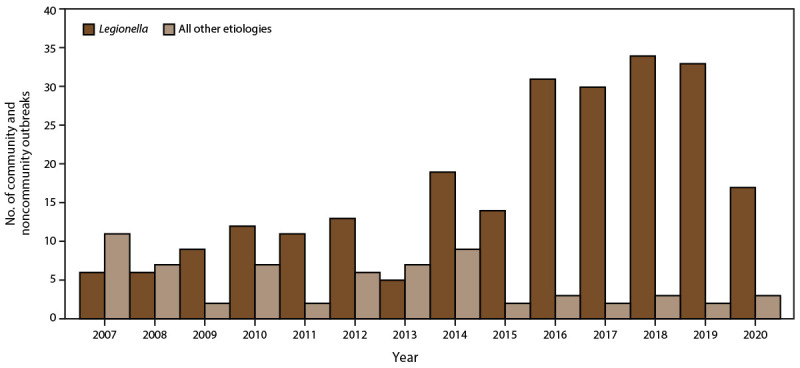
Number of reported drinking water-associated outbreak community and noncommunity water settings,* by *Legionella* compared with all other etiologies — Waterborne Disease and Outbreak Surveillance System, United States, 2007–2020 * N = 306 water settings.

Drinking water treatment, regulations, and public health programs reduce the risk for exposure to drinking water pathogens, chemicals, and toxins in the United States. Recent estimates of waterborne infectious illness and health care cost effects in the United States have revealed that biofilm-associated pathogens, *Legionella* and NTM, have emerged as the predominant causes of hospitalizations and deaths from waterborne and drinking water-related disease (*3*). However, NTM infections are not nationally notifiable diseases and cases and outbreaks might remain undetected (*3*). Furthermore, during 2015–2020, *Legionella*-associated outbreaks continued to increase and were the leading cause of nationally reported drinking water-related outbreaks, hospitalizations, and deaths. This trend was primarily influenced by the increasing number and proportion of *Legionella*-associated outbreaks linked with community and noncommunity water systems reported to NORS during the study period (Figures 4 and 5) (*6*). In addition, *Legionella* was implicated in all lodging and nearly all (n = 111; 98%) health care-associated biofilm-related outbreaks. Furthermore, *Legionella*-associated outbreaks in health care settings resulted in approximately two thirds (n = 364; 65%) of hospitalizations and three fourths (n = 73; 85%) of deaths reported during this period. These findings highlight the severity of *Legionella* infection in the health care setting (*13*). *Legionella* also was reported for the first time to NORS as the cause of three outbreaks in private residences. *Legionella*-associated outbreaks in private residences is an emerging concern. Additional data are needed to better characterize the role of premise plumbing systems in private homes as a potential source of exposure to *Legionella* and Legionnaires' disease outbreaks (*14*). These outbreaks illustrate the importance of effective regulations, water management programs, and public health prevention programs that include communications to reduce the risk for biofilm pathogen growth and spread in public drinking water systems, building water systems, and private homes (*4*,*15*,*16*).

Enteric illness outbreaks represent 11% (n = 24) of the outbreaks, approximately 60% of the cases during this reporting period. Settings varied widely, including mobile home parks, lodging, amusement parks, farms, camps, and private residences. Enteric illness outbreaks associated with norovirus, *Shigella*, *Campylobacter*, or multiple etiology outbreaks were primarily associated with individual or private and community water systems. One outbreak of norovirus and enteropathogenic *E. coli* that resulted in 693 (32%) cases occurred in an amusement park setting because of an overly pumped, improperly constructed well with chronically inadequate disinfection. Wells also were identified as the water source when reported, regardless of water system type (i.e., community or individual or private) in nearly all (n = 13; 93%) enteric illness drinking water outbreaks. No disinfection was reported in nearly 75% (n = 11) of these outbreaks when water treatment was known, underscoring the importance of proper well construction, location (i.e., under the influence of surface water or proximity to wastewater disposal system), operation, and maintenance (*17*–*20*).

Understanding and communicating contributing factors related to waterborne outbreaks can lead to improved outbreak prevention, response, and communication practices (*21*,*22*). Most drinking water-associated outbreaks have multiple contributing factors, and the most frequently reported types vary between *Legionella*-associated and enteric illness outbreaks. For example, premise plumbing or point of use is the most cited contributing factor type for *Legionella*-associated outbreaks, whereas water source is most cited for enteric illness outbreaks. Furthermore, most *Legionella*-associated outbreak investigations are prompted by cases associated with premise plumbing systems. As a result, premise plumbing contributing factors (e.g., inadequate disinfection or *Legionella*-promoting water temperatures) are frequently identified. Determining the potential role of other upstream contributing factor types (e.g., water distribution systems) might be difficult. Whereas enteric illness investigations outbreaks frequently result from upstream contributing factors (e.g., disinfection failure or well or groundwater contamination) and can result in many cases of illness. The observed range of biofilm and enteric drinking water pathogen contributing factors illustrates the complexity of drinking water-related disease prevention and the need for water source-to-tap prevention strategies (*16*,*20*,*23*).

## Limitations

The findings in this report are subject to at least four limitations. First, reporting to NORS is voluntary, and surveillance, outbreak investigation, and reporting capabilities vary by jurisdiction. Outbreak surveillance data might not represent the characteristics of all outbreaks and likely underestimate the actual occurrence of outbreaks. Therefore, NORS data should not be used to estimate the actual number of outbreaks. Reports of investigated outbreaks vary, and data are limited to what is available and reported by jurisdictions. Second, *Legionella*-associated outbreak investigations can continue for years, with new cases of illness occurring after extended periods; jurisdictions might not report to NORS until conclusion of the investigation. Third, the COVID-19 pandemic might have affected jurisdictions’ ability to report waterborne disease outbreaks during 2019–2020. Finally, contributing factors were not available for 70 (33%) drinking water-associated outbreaks, and water treatment was unknown for 49 (23%) drinking water-associated outbreaks. Furthermore, certain *Legionella* and biofilm-associated pathogen outbreak contributing factors that were self-reported as distribution factor types by users align more closely with premise or point-of-use factor types, possibly resulting in misclassification bias. *Legionella* species in water systems was frequently reported as a contributing factor and does not provide insight into factors that led to *Legionella* growth and spread within the water system.

## Future Directions

NORS was updated substantially (CDC 52.14 Form; https://www.cdc.gov/nors/forms.html) in January 2023. The update streamlined the environmental sampling results section, added a section about outbreaks caused by *Legionella* and other biofilm-associated pathogens, revised the contributing factors section, and created a new interventions section to capture interventions that were recommended or implemented to help stop outbreaks. Future analyses can leverage these NORS updates to improve understanding of biofilm-associated outbreaks, contributing factors, interventions, and water management program failures. Previous studies have highlighted the importance of examining data from *Legionella*-associated outbreak investigations, including water management program failures which can lead to improved water management practices (*13*,*21*,*22*). Both the Veterans Health Administration and the Centers for Medicare and Medicaid Services have directives requiring the implementation of water management programs in specific health care facilities to reduce the risk related to biofilm-associated pathogens, including *Legionella* (*24*,*25*).

Improvements in biofilm-associated pathogen surveillance and outbreak reporting could lead to greater outbreak detection and guide disease prevention strategies. Recent efforts to estimate the illness and health care cost impacts of waterborne disease in the United States have revealed that *Legionella*, NTM, and biofilm-associated pathogens have emerged as the predominant causes of hospitalizations and deaths from waterborne diseases, including those linked to drinking water exposures (*3*). The Council of State and Territorial Epidemiologists has a standardized case definition for extrapulmonary NTM infections (opportunistic infections of wounds, soft tissue, or joints) to ensure consistency in reporting, and to identify outbreaks (*26*). NTM case reporting, and definitions vary across state and local public health departments, with certain departments reporting pulmonary and extrapulmonary, extrapulmonary only, or NTM site and species not specified (*27*–*29*). Health departments could consider making extrapulmonary NTM infections reportable within their jurisdictions (*26*). In addition, active population-based NTM surveillance, currently occurring in certain jurisdictions, will provide important data for monitoring the illness and health care cost impacts of disease, identifying affected populations, and informing public health prevention strategies (*26*,*28*).

## Conclusion

Public health surveillance is essential to monitor trends in waterborne disease and detect outbreaks related to drinking water exposures. During 2015–2020, public health officials from 28 states reported 214 outbreaks associated with drinking water. These outbreaks resulted in at least 2,140 cases of illness, 563 hospitalizations (26% of cases), and 88 deaths (4% of cases). *Legionella*-associated outbreaks increased in number and were the leading cause of drinking water-associated outbreaks reported to NORS during the surveillance period, including hospitalizations and deaths. Primary prevention of *Legionella*-associated outbreaks through biofilm control and water management remains critical in health care and nonhealth care settings. Outbreaks of enteric illness primarily linked to wells represented over half of the cases during the reporting period, underscoring the importance of disease prevention efforts related to groundwater. The emergence of biofilm-associated pathogens as the primary influence of drinking water-associated outbreaks, along with the risk for enteric illness outbreaks capable of causing large numbers of cases, highlights the need for agile waterborne disease surveillance, prevention, and outbreak response programs. Drinking water source-to-tap partnership and prevention strategies are critical to addressing the emerging issue of biofilm-associated disease and to guide holistic biofilm pathogen prevention strategies. Drinking water regulations and water management programs are essential to controlling pathogens in drinking water to prevent drinking water-associated outbreaks.
